# Health-related quality of life on tele-monitoring for users with pacemakers 6 months after implant: the NORDLAND study, a randomized trial

**DOI:** 10.1186/s12877-018-0911-3

**Published:** 2018-09-21

**Authors:** Antonio Lopez-Villegas, Daniel Catalan-Matamoros, Remedios Lopez-Liria, Terje Enebakk, Hilde Thunhaug, Knut Tore Lappegård

**Affiliations:** 1 Social Involvement of Critical and Emergency Medicine, CTS-609 Research Group, Hospital de Poniente, Almeria, Spain; 2 Division of Medicine, Nordland Hospital, Bodø, Norway; 3 Institute of Clinical Medicine, Faculty of Health Sciences, University of Tromsø, Tromsø, Norway; 4 Department of Journalism and Communication, Universidad Carlos III de Madrid, Calle Madrid 133, 28903 Getafe, Madrid, Spain; 5 Health Sciences CTS-451 Research Group, University of Almeria, Almería, Spain; 6 Nursing Science, Physiotherapy and Medicine, Faculty of Health Sciences, University of Almeria, Almería, Spain

**Keywords:** Health-related quality of life, Older adults, Pacemaker follow-up, Randomized study, Remote monitoring, Telemedicine

## Abstract

**Background:**

With an ageing population and widening indications for pacemakers implantation, the number of persons carrying an implant is steadily increasing. The routine follow-up is thus a heavy burden for the respective NHS as well as for the patients and their relatives. Most of them of the studies have been performed in densely populated areas and nearby to the hospital. It is thus unknown whether these results could be applied also in rural areas such as Northern Norway with a more scattered population. The aim of this study was to assess the effectiveness of tele-monitoring (TM) in patients with pacemakers regarding reliability, safety and health-related quality of life, compared to traditional follow-up in outpatient clinic in a setting where geographical effects could possible influence the results.

**Methods:**

The NORDLAND study is a controlled, randomized, non-masked clinical trial in pacemaker patients, with data collection carried out during the pre-implant stage and after 6 months. Between August of 2014 and November of 2015, 50 patients were assigned to either a tele-monitoring group (*n* = 25) or a conventional hospital monitoring (HM) group (*n* = 25). The *EuroQol-5D* (EQ-5D) utilities and visual analogue scale (VAS) and the Minnesota Living with Heart Failure Questionnaire *(MLHFQ)* were used to measure Health-Related Quality of Life. Baseline characteristics and number of hospital visits were also analyzed.

**Results:**

The baseline characteristics of the two study groups were similar for EQ-5D utilities (TM:0.81; HM:0.76; *p* = 0.47), EQ-5D VAS (TM: 64.00; HM:64.88; *p* = 0.86) and the *MLHFQ* (TM:20.20; HM:28.96; *p* = 0.07). At the 6 month follow-up, there were no significant differences between the groups in EQ-5D utilities (TM: 0.81; HM: 0.76; *p* = 0.54) and EQ-5D VAS scores (TM: 72.71; HM: 59.79; *p* = 0.08). The *MLHFQ* score was improved in both groups (TM: -4.40; HM: -15.13; *p* <  0.001). The number of in-office visits was similar in both groups (TM: 1.24 vs HM: 1.12; *P* = 0.30).

**Conclusions:**

The NORDLAND trial shows that HRQoL is improved after implant in both groups. Without significant differences with regards to effectiveness and safety. In addition, provides a scientifically rigorous method to the field of HRQoL evaluations in patients with pacemakers.

**Trial registration:**

ClinicalTrials.gov NCT02237404, September 11, 2014.

## Background

International guidelines of professional practice advises that users with pacemakers (PM) must be monitored in periods between 3 and 12 months [1, 2]. Every hospital visit usually involves an assessment of pacemakers function, cardiovascular events and an analysis of patient physical status and, whether is necessary the medication is modified and/or the device is re-configured again [1, 3]. With an ageing population and widening indications for PM implantation, the number of persons carrying an implant is steadily increasing. The routine follow-up is thus a heavy burden for the respective national health services [4] as well as for the pacemakers users and their caregivers [5, 6].

Remote monitoring or tele-monitoring (TM) systems have potential advantages such as early detection of cardiovascular events and early response to technical problems of the device or changes in the patient’s clinical status [7–10]. TM may be a potential alternative to help reduce the number of in-office visits to hospital, thereby optimizing medical resources [11]. Along last decade, several studies have showed that TM is as effective as traditional follow-up in hospital [2, 10–13]. In addition, the number of unscheduled visits to the hospital for assistance at the emergency services and hospitalizations can be reduced [4, 14]. In the last decade a limited number of studies have shown results on efficiency [4, 7, 15, 16] particularly based on patient-reported outcomes such as HRQoL [10–12, 15–18]. On the other part, a study published recently affirms that telemedicine is not significantly more effective than usual care on mental and physical Health-Related Quality of Life (HRQoL) [19].

However, the majority of these studies have not been randomized, but have based their allocation to mode of follow-up to patient or observer preference. Furthermore, several of them have been performed in densely populated areas with most of the patients living only a short distance to the following centre. It is thus unknown whether these results apply also in a randomized study and in rural areas such as Northern Norway with a more scattered population.

Regarding previous paragraph, there is no international standard for defining rural areas, and the existing ones may vary even within a same country [20, 21]. Previous publications indicate that the healthcare needs of patients living in rural areas usually be different from those in urban areas, and patients living in rural areas often they tend to have problem access to healthcare [22]. Nordland county (Norway) has a scattered population of 6,3 inhab/sq. km (for comparison Canada has 3,7, USA 34,8 and United Kingdom 274 inhab/sq. km) [20, 23]. Although a large fraction of the population in Nordland county (Norway) lives in cities, the cities are small and there is still a significant proportion of inhabitants living in communities with less than 2500 individuals and at distances a long way from the hospital.

The purpose of the present study was to assess the effectiveness of TM in patients with pacemakers regarding reliability, safety and health-related quality of life, compared to traditional follow-up in outpatient clinic in a setting where geographical effects could possible influence the results.

## Methods

### Design

Controlled, randomized, non-masked clinical trial with patient assignment to conventional follow-up in the hospital or tele-monitoring follow-up by electronic data transmission with 6 months monitoring from the date of implantation.

The randomization process was performed as follows: A person unrelated to the study prepared a total of 50 sealed envelopes, 25 which included at note reading “tele-monitoring” and 25 with a note reading “hospital monitoring”. The envelopes were thoroughly mixed and numbered from 1 to 50. When a patient had accepted the invitation to participate in the study and signed the informed consent he was given a consecutive study number and allocated to follow-up in accordance with the specification included in the corresponding envelope. Thus, the investigators had no knowledge of or influence on the randomization result prior to inclusion.

### Environment

The study was carried out in Nordland Hospital, Bodø, Norway, a hospital with a pacemaker center that serves a population of 170,000 inhabitants and performs around 80–90 pacemakers implants per year.

### Participants and selection

All patients who were scheduled for implant with a pacemaker between August of 2014 and October of 2015 and met all the inclusion criteria (age 18 years or older, ability to give informed consent and to operate the home monitor, life expectancy > 1 year) and none of the exclusion criteria (scheduled for implantable cardioverter-defibrillator (ICD) or cardiac resynchronization therapy (CRT), participation in other trials) were invited to participate. A total of 76 patients were screened and 50 patients were included and randomized to either telemonitoring (TM, *n* = 25) or hospital monitoring (HM, *n* = 25). No changes to methods were performed after trial commencement.

### Devices

According to their diagnosis, patients received either a single (VVIR) or a dual chamber pacemaker (DDDR). Patients assigned to tele-monitoring received either a Biotronik Estella SR-T/ DR-T or a Biotronik Evia SR-T/ DR-T. Patients assigned to hospital monitoring received either one of the aforementioned Biotronik pacemakers, a St Jude Medical Endurity SR/DR or a Sorin Reply 200 SR/DR.

Home monitoring was performed through the Biotronik Home Monitoring® system, an internet-based TM service for users with Biotronik implantable heart devices. Biotronik devices equipped with Home Monitoring (T-devices) have additional storage capacity and contain a small RF-antenna for wireless communication and data transmission from the implant to a wireless patient device, the CardioMessenger. Home Monitoring has no negative effects on devices’ battery life [24].

Every night, the CardioMessenger automatically collects and transmits important, encrypted health information to the Biotronik service center using the global network of T-Mobile and its partners (GPRS). The transmitted patient data is collected, automatically analyzed and filtered at the Biotronik Home Monitoring Service Center, according to patients‘needs as defined by their physician. Health and system-related issues are ranked and marked in order of importance. All event and trend reports can be accessed and reviewed on a protected online platform. Furthermore, according to pre-set definitions, the physician can receive automatic warnings (e.g. by e-mail or text message) concerning safety issues such as premature battery depletion, lead fracture etc.

### Primary outcome measures

HRQoL of the users with pacemakers was assessed through the Norwegian version of the utility scores of the EuroQol-5D (EQ-5D) [25] from − 1 [the worst state of health] to 1 [the best state of health], and the self-rated HRQoL was analyzed through of the EQ-5D Visual Analogue Scale (EQ-5D-VAS), from 0 [worst imaginable health state or death] to 100 [perfect health]).

### Secondary outcome measures

To measure the effects of symptoms, functional limitations and psychological reactions commonly associated with heart failure or its treatment on an individual’s quality of life, the Norwegian validated version of the MLHFQ. It consists of 21 questions regarding patients’ perception of the effects of HF on their daily lives; the score for each question ranges from 0 to 5, producing a total score of 0 to 105, with a higher score indicating a poorer HRQoL [26–29].

### Other outcome measures

Differences between TM and HM groups, in regards to: 1) Cardiovascular events (AE), including exitus, percutaneous coronary intervention, angina and lead dislodgement 2) monitoring visits and transmissions from home vs. hospital and 3) changes of medication and pacemakers reprogramming.

### Follow-up

Patients were given 3 interviews (on the day of the pacemakers implant, and at 1 and 6 months after the implant) in which the corresponding questionnaires were administered. These interviews were carried out in a personalized way during hospitalization or by telephone interviews after discharge.

To ensure that the sample size was representative, due to the limited capacity of patient enrolment in the hospitaI, the following formula was used: $\boldsymbol{n}=\frac{\boldsymbol{N.}{\boldsymbol{Z}}^{\mathbf{2}}.\boldsymbol{p}\left(\mathbf{1}-\boldsymbol{p}\right)}{\left(\boldsymbol{N}-\mathbf{1}\right){\boldsymbol{e}}^{\mathbf{2}}+{\boldsymbol{Z}}^{\mathbf{2}}.\boldsymbol{p}\left(\mathbf{1}-\boldsymbol{p}\right)}$ Thus, from a target population of *n* = 70, assuming a standard deviation of Z = 1.96, with a margin of error e = 0.10, a proportion of *p* = 0.5, and a 10% of possible losses, we got a size sample necessary of *n* = 45. We did not have any prior information on the value that was expected to be found. For this reason, 50% has been used as the statistical power (*p* = 0.5) meaning that, if there was any effect, it would be detected at the 50% of the total events.

### Statistical analysis

Continuous variables were assessed as mean ± standard deviation. Categorical data in both groups were compared using difference in proportions test (binomial method) or Chi-Square test or Fisher exact test, when was appropriated. Differences between both groups were also assessed in the pre-specified endpoints using the difference in means or proportions tests and Wilcoxon signed ranks test for *Minnesota Living with Heart Failure Questionnaire*. Results are presented, including the corresponding 95% confidence intervals (95% CI). All statistical analyses were performed with SPSS (SPSS Institute, Inc., Chicago, IL, USA) statistical software.

## Results

### Participants

**Fig. 1:**
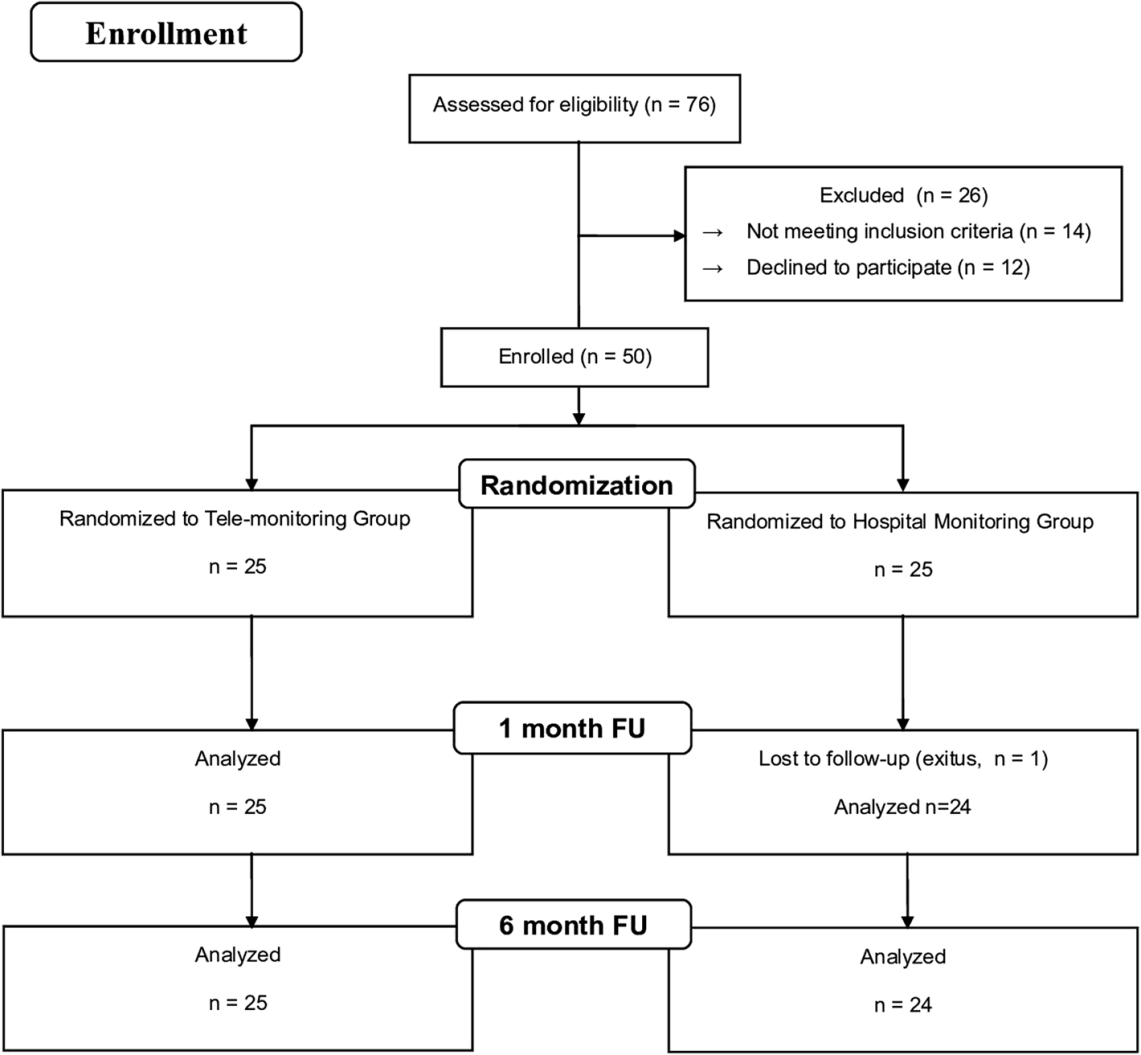
Flow (CONSORT) diagram of the study

Between August of 2014 and October of 2015, 50 patients (mean age: 75 ± 12 years, 48% women) were enrolled in the NORDLAND study, of which 25 were included in the TM group and 25 in the HM group (Fig. 1). Dizziness was the most common symptom, and the patients were most commonly referred from the hospital’s own cardiology ward or from other local hospitals. The most common pacing indication was sick sinus syndrome (48%) followed by atrioventricular block (40%). In 88% of the cases, DDDR pacemakers were used. Hypertension was the most frequent comorbidity (64%). None of the baseline characteristics, including HRQoL were different between the two groups (Table 1).

**Table 1:** Patients’ demographic and clinical characteristics at baseline

	All (*n* = 50)	Groups		*P*-value
		TM (*n* = 25)	HM (*n* = 25)	
Age (mean) ± SD	74.84 ± 11.8	73.68 ± 14.2	76.00 ± 8.8	0.68
Men (mean)	26 (52.0)	13 (52.0)	13 (52.0)	1.00
MLHFQ [95CI]	24.58 [19.30;29.86]	20.20 [14.48;25.92]	28.96 [19.97–37.95]	0.07
EQ5D utilities [95CI]	0.78 [0.72;0.85]	0.81 [0.74;0.87]	0.76 [0.64;0.88]	0.47
Pacing indication *(%)*				
Sick sinus syndrome	24 (48.0)	12 (48.0)	12 (48.0)	0.65
Atrioventricular block	20 (40.0)	11 (44.0%)	9 (36.0)	
Chronic AF with Bradycardia	6 (12.0)	2 (8.0)	4 (16.0)	
Disease manifestations *(%)*				
Syncope	14 (28.0)	8 (32.0)	6 (24.0)	0.81
Dizziness	25 (50.0)	12 (48.0)	13 (52.0)	
Dyspnoea	11 (22.0)	5 (20.0)	6 (24.0)	
Service derivated (%)				
Emergency dept.	3 (6.0)	1 (4.0)	2 (8.0)	0.51
Cardiology ward	14 (28.0)	5 (20.0)	9 (36.0)	
Primary healthcare	4 (8.0)	2 (8.0)	2 (8.0)	
Other hospitals	29 (58.0)	17 (68.0)	12 (48.0)	
Stimulation (%)				
DDDR	44 (88.0)	23 (92.0)	21 (84.0)	0.33
VVIR	6 (12.0)	2 (8.0)	4 (16.0)	
Comorbidities (%)				
Dislipidemia	27 (54.0)	13 (52.0)	14 (56.0)	0.50
Obesity (BMI > 30)	1 (2.0)	0 (0.0)	1 (4.0)	0.50
Tachyarrhythmia	18 (36.0)	7 (28.0)	11 (44.0)	0.19
Hypertension	32 (64.0)	17 (68.0)	15 (60.0)	0.38
Diabetes Mellitus	6 (12.0)	0 (0.0)	6 (24.0)	0.01
Other comorbidities (%)				
None	18 (36.0)	11 (44.0)	7 (28.0)	0.39
Others	10 (20.0)	6 (24.0)	4 (16.0)	
Coronary heart diseases	22 (44.0)	8 (32.0)	14 (56.0)	
Pharmaceutical treatment (%)				
Antiaggregants	18 (36.0)	8 (32.0)	10 (40.0)	0.38
Anticoagulants	25 (50.0)	10 (40.0)	15 (60.0)	0.13
Antiarrhythmics	18 (36.0)	7 (28.0)	11 (44.0)	0.19
Antihypertensives	32 (64.0)	18 (72.0)	14 (56.0)	0.19

*N* = 50 (Telemonitoring group: 25; Hospital Monitoring group: 25). Values are expressed as means or proportions. 95CI: 95% confidence interval of means; *AF* atrial fibrillation, *BMI* body mass index, *EQ5D* EuroQoL-5D, *DDD* bicameral pacemaker with two electrodes placed in the atrium and in the ventricle with the ability to modulate frequency of stimulation, *MLHFQ* Minnesota Living with Heart Failure, *VVIR* unicameral pacemaker with an electrode in the ventricle with the ability to modulate frequency of stimulation

### Health-related quality of life basal analysis

In basal analysis patients had a mean score of 0.78 (95%CI:0.72;0.85) for EQ5D utilities and 64.83 (95% CI:56.74;70.14) for EQ5D VAS, which was increased to 0.81 (95%CI: -0.75;0.87) and 67.80 (95%CI: 62.49;73.10) respectively at 1 month after PM implantation (Table 2). Changes between baseline and 1 month EQ5D utilities and VAS were not statistically significant (*p* = 0.81 and *p* = 0.20, respectively). TM and HM groups showed no significant differences in EQ5D utilities and VAS scores between enrollment and at month (Table 2), nor at any EQ5D utilities dimensions. Regarding MLHFQ, the patients had a mean score at baseline of 24.58 (95%CI:19.30;29.86). This improved significantly to 16.55 (95%CI:11.40–21.70) at the 1 month assessment (*p* <  0.05). There were no significant differences in MLHFQ scores between TM and HM groups (20.20 vs. 28.96; *p* = 0.07) at baseline or at 1 month of follow-up (18.64 vs. 14.38, *p* = 0.59) (Table 3).

**Table 2:** Health-related quality of life at 6 months of follow-up

EQ-5D Utilities		Month 0				Month 1				Month 6			
		TM n (%)	HM n (%)	Total n (%)	p	TM n (%)	HM n (%)	Total n (%)	p	TM n (%)	HM n (%)	Total n (%)	p
Mobility	No problems	15 (60.0)	12 (48.0)	*27 (54.0)*	*0.29*	16 (64.0)	*14 (58.3)*	30 (61.2)	0.46	17 (68.0)	16 (66.7)	33 (67.3)	0.58
	Some problems	10 (40.0)	13 (52.0)	*23 (46.0)*		9 (36.0)	*10 (41.7)*	19 (38.8)		8 (32.0)	8 (33.3)	16 (32.7)	
	Extreme problems	0 (0.0)	0 (0.0)	0 (0.0)		0 (.0)	0 (0.0)	0 (0.0)		0 (0.0)	0 (0)	0 (0.0)	
Self-care	No problems	22 (88.0)	22 (88.0)	*44 (88.0)*	*0.67*	23 (92.0)	*22 (91.7)*	45 (91.8)	0.68	21 (84.0)	23 (95.8)	44 (89.8)	0.19
	Some problems	22 (88.0)	22 (88.0)	*6 (12.0)*		2 (8.0)	*2 (8.3)*	4 (8.2)		4 (16.0)	1 (4.2)	5 (10.2)	
	Extreme problems	0 (0.0)	0 (0.0)	0 (0.0)		0 (0.0)	0 (0.0)	0 (0.0)		0 (0.0)	0 (0.0)	0 (0.0)	
Usual activities	No problems	16 (64.0)	14 (56.0)	*30 (60.0)*	*0.84*	13 (52.0)	*13 (54.2)*	26 (53.1)	0.55	18 (72.0)	12 (50.0)	30 (61.2)	0.22
	Some problems	8 (32.0)	10 (40.0)	18 (36.0)		12 (48.0)	*11 (45.8)*	23 (46.9)		7 (28.0)	11 (45.8)	18 (36.7)	
	Extreme problems	1 (4.0)	1 (4.0)	2 (4.0)		0 (0.0)	0 (0.0)	0 (0.0)		0 (0.0)	1 (4.2)	1 (2.0)	
Pain / discomfort	No problems	11 (44.0)	14 (56.0)	*25(50.0)*	*0.08*	13 (52.0)	*15 (62.5)*	28 (57.1)	0.71	13 (52.0)	12 (50.0)	25 (51.0)	0.87
	Some problems	14 (56.0)	8 (32.0)	*22 (44.0)*		10 (40.0)	*8 (33.3)*	18 (36.7)		9 (36.0)	10 (41.7)	19 (38.8)	
	Extreme problems	0 (0.0)	3 (12.0)	*3 (6.0)*		2 (8.0)	*1 (2.0)*	3 (6.1)		3 (12.0)	2 (8.3)	5 (10.2)	
Anxiety / depression	No problems	16 (64.0)	16 (64.0)	*32 (64.0)*	*0.62*	18 (72.0)	*19 (79.2)*	37 (75.5)	0.58	16 (64.0)	19 (79.2)	35 (71.4)	0.50
	Some problems	9 (36.0)	9 (36.0)	*18 (36.0)*		6 (24.0)	*5 (20.8)*	11 (22.4)		7 (28.0)	4 (16.7)	11 (22.4)	
	Extreme problems	0 (0.0)	0 (0.0)	0 (0.0)		1 (4.0)	*0 (0.0)*	1 (2.0)		2 (8.0)	1 (4.2)	3 (6.1)	
	TOTAL [95CI]	0.81 [0.74;0.87]	0.76 [0.64;0.88]	0.78 [0.72;0.85]	*0.47*	0.79 [0.70;0.88]	0.83 [0.74;0.92]	0.81 [0.75;0.87]	0.49	0.82 [0.69;0.93]	0.76 [0.65;0.87]	0.79 [0.71;0.86]	0.54
EQ-5D VAS													
	TOTAL [95CI]	64.00 [55.77;72.23]	64.88 [51.69;74.07]	64.83 [56.74;70.14]	0.86	69.38 [60.82;76.38]	66.96 [59.17;74.75]	67.80 [62.49;73.10]	0.71	72.71 [65.58;79.83]	59.79 [49.44;70.14]	66.74 [60.46;73.01]	0.08

*TM* tele-monitoring group, *HM* hospital monitoring group; N (month 0) = 50 (TM group: 25; HM group: 25). N (month 1) =49 (TM group: 25; HM group: 24). N (month 6) =49 (TM group: 25; HM group: 24). Values are expressed as means [95CI: 95% confidence interval of means]. EQ5D: EuroQoL-5D; VAS: Visual Analog Scale

**Table 3:** Minnesota Living with Heart Failure Questionnaire (MLHFQ) scores at 6 months

	Month 0			Month 1			Month 6		
	TM *N* = 25	HM *N* = 25	p	TM *N* = 25	HM *N* = 24	p	TM *N* = 25	HM *N* = 24	p
Swelling in ankles, legs	0.84	1.00	0.72	0.50	0.58	0.88	0.25	0.42	0.57
Rest during the day	1.28	2.08	0.13	1.29	1.08	0.75	1.08	0.88	0.69
Walking and climbing stairs	0.96	1.92	0.09	0.96	0.54	0.41	0.63	0.63	0.96
Working around the house	1.20	2.36	0.03	0.96	1.25	0.61	0.50	1.13	0.18
Going away from home	0.28	1.44	0.01	0.92	0.21	0.06	0.79	0.83	0.92
Sleeping	0.84	1.32	0.27	0.71	0.71	0.94	0.58	0.50	0.83
Doing things with friends or family	0.88	1.96	0.04	1.04	0.71	0.42	1.04	0.83	0.68
Working to earn a living	0.40	0.72	0.23	0.50	0.08	0.14	0.00	0.04	0.32
Recreational pastimes	1.24	1.76	0.23	1.29	0.88	0.39	1.21	1.17	0.92
Sexual activities	0.32	0.68	0.31	0.25	0.42	0.52	0.71	0.67	0.96
Eat less of the food liked	0.60	1.00	0.21	0.71	0.42	0.36	0.50	0.17	0.34
Shortness of breath	2.44	2.48	0.99	1.46	1.04	0.32	1.29	1.50	0.60
Fatigue, tireness, low on energy	2.36	3.20	0.08	1.54	1.38	0.76	1.38	1.38	0.97
Stay in the hospital	1.48	1.76	0.58	1.00	1.38	0.55	0.58	0.38	0.60
Costing money for medical care	0.60	0.80	0.50	0.71	0.42	0.19	0.25	0.13	0.85
Side effects from medication	0.44	0.68	0.47	0.13	0.83	0.06	0.54	0.79	0.53
Feeling burdensome	0.32	0.76	0.19	0.46	0.08	0.12	0.21	0.21	0.71
Feeling a loss of self control	0.80	0.76	0.95	0.54	0.46	0.72	0.42	0.38	0.92
Worry	1.00	0.88	0.64	0.79	0.67	0.81	0.67	0.38	0.41
Difficulty remembering & concentrating	1.16	0.96	0.53	1.00	0.75	0.59	0.92	0.54	0.37
Feeling depressed	0.44	0.76	0.37	0.71	0.50	0.72	0.54	0.29	0.50
TOTAL [95CI]	20.20 [14.48–25.92]	28.96 [19.97–37.95]	0.07	18.64 [11.01–26.27]	14.38 [6.99–21.76]	0.59	15.80 [7.18–24.42]	13.21 [5.86–20.56]	0.97

TM: Tele-monitoring group; HM: Hospital monitoring group; N (month 0) = 50 (TM group: 25; HM group: 25). N (month 1) = 49 (TM group: 25; HM group: 24). N (month 6) = 49 (TM group: 25; HM group: 24). Items values are expressed as means. Total values are expressed as means [95CI: 95% confidence interval of means]

### Cardiovascular adverse events and workload

After 6 months of follow-up, 6% of patients had experienced at least one AE (TM: 6%, HM: 4.2%; *p* = 0.40) (Table 4). After PM implantation, 38.2% of patients were hospitalized at least once (TM: 42%, HM: 33.4%; *p* = 0.55). The main causes of hospitalization were due to coronary problems (TM, 16% vs. HM, 20.8%) and PM dysfunction (TM, 12% vs. HM, 0%), however no significant differences between the groups were found. One patient (HM group) died for non-cardiovascular causes. As for workload, a mean of 1.20 ± 0.50 in-hospital visits per patient were registered during the follow-up period (TM:1.24 vs. HM:1.17; *p* = 0.26).

**Table 4:** Follow-up information at 6 months

	Total N (%)	Tele-monitoring N (%)	Hospital Monitoring N (%)	*P*-value
Number of transmissions from consultation room				
0	0 (0.0)	0 (0.0)	0 (0.0)	0.26
1	41 (83.7)	21 (84.0)	20 (83.3)	
2	6 (12.2)	2 (8.0)	4 (16.7)	
3	2 (4.1)	2 (8.0)	0 (0.0)	
Transmissions from patients home				
0	29 (59.2)	5 (20.0)	24 (100)	< 0.001
3–5	15 (30.6)	15 (60.0)	0 (0.0)	
6–8	5 (10.2)	5 (20.0)	0 (0.0)	
Extra transmissions from patients home				
0	45 (91.8)	21 (84.0)	24 (100)	0.12
1	1 (2.0)	1 (4.0)	0 (0.0)	
3	3 (6.2)	3 (12.0)	0 (0.0)	
Cardiovascular adverse events				
None	46 (93.9)	23 (92.0)	23 (95.8)	0.40
PCI	1 (2.0)	1 (4.0)	0 (0.0)	
Angina	1 (2.0)	0 (0.0)	1 (4.2)	
Lead dislodgement ×2	1 (2.0)	1 (2.0)	0 (0.0)	
Changes of medication				
0	33 (67.3)	17 (68.0)	16 (66.7)	0.11
1	7 (14.3)	5 (20.0)	2 (8.3)	
2	3 (6.1)	1 (4.0)	2 (8.3)	
3	4 (8.2)	0 (0.0)	4 (16.7)	
4	2 (4.1)	2 (8.0)	0 (0.0)	
Changes of pacemakers reprogramming				
0	34 (69.4)	16 (64.0)	18 (75.0)	0.34
1	13 (26.5)	7 (28.0)	6 (25.0)	
2	2 (4.1)	2 (8.0)	0 (0.0)	
Hospitalizations days after implant				
0	30 (61.2)	14 (56.0)	16 (66.7)	0.55
1	14 (28.6)	7 (28.0)	7 (29.2)	
2	4 (8.2)	3 (12.0)	1 (4.2)	
5	1 (2.0)	1 (2.0)	0 (0.0)	
Days hospitalizated				
0	30 (61.2)	14 (56.0)	16 (66.7)	0.54
1–5	12 (24.5)	6 (24.0)	6 (25.1)	
6–10	4 (8.1)	2 (8.0)	2 (8.4)	
+ 10	3 (6.0)	3 (12.0)	0 (0.0)	
Reasons for hospitalization				
None	30 (61.2)	14 (56.0)	16 (66.7)	0.37
Others	6 (12.3)	3 (12.0)	3 (12.5)	
Cancer	1 (2.0)	1 (4.0)	0 (0.0)	
Coronary problems	9 (18.4)	4 (16.0)	5 (20.8)	
PM disfuction	3 (6.1)	3 (12.0)	0 (0.0)	

*TM* tele-monitoring group, *HM* hospital monitoring group, N (month 6) = 49 (TM group: 25; HM group: 24). Items values are expressed as N (%). *PCI* percutaneous coronary intervention, *PM* pacemakers

### Health related quality of life results at 6 months

At 6 months, the EQ5D utilities score (Table 5) was increased to 0.79 (95%CI: 0.71;0.86; *p* = 0.54), 0.01 points more than baseline and 0.02 points less than the scores reached in month 1 (95% CI: -0.16;0.10; *p* = 0.64). On the EQ-5D VAS, the mean score reached 66.74 points, 1.91 points higher than in basal analysis (relative increase of 2.86%; *p* = 0.42) and 1.06 points less than the obtained in month 1 after implant (relative decrease of 1.59%; *p* = 0.72).

**Table 5:** Differences in Health-Related Quality of Life at 6 months of follow-up

	All				Tele-Monitoring				Hospital Monitoring			
	Month 0	Month 6	Differences	p	Month 0	Month 6	Differences	p	Month 0	Month 6	Differences	p
Health Related Quality of Life - Specific												
MLHFQ [95CI]	24.58 [19.30–29.86]	14.53 [9.04–20.02]	−9.65 [4.03;15.27]	< 0.001	20.20 [14.48–25.92]	15.80 [7.18–24.42]	−4.40 [−3.08;11.88]	0.04	28.96 [19.97–37.95]	13.21 [5.86–20.56]	−15.13 [6.73;23.52]	< 0.001
Health Related Quality of Life - General												
EQ5D VAS [95CI]	64.83 [56.74;70.14]	66.74 [60.46;73.01]	1.91 [−10.41;4.37]	0.42	64.00 [55.77;72.23]	72.71 [65.58;79.83]	8.71 [−16.79;0.54]	0.07	64.88 [51.69;74.07]	59.79 [49.44;70.14]	5.09 [−8.97;19.13]	0.46
EQ5D utilities [95CI]	0.78 [0.72;0.85]	0.79 [0.71;0.86]	0.01 [−0.08;0.12]	0.71	0.81 [0.74;0.87]	0.81 [0.69;0.93]	0.00 [−0.14;0.12]	0.92	0.76 [0.64;0.88]	0.76 [0.65;0.87]	0.00 [−0.07;0.13]	0.50

N (month 1) =49 (TM group: 25; HM group: 24). N (month 6) =49 (TM group: 25; HM group: 24). Values are expressed as means [95CI: 95% confidence interval of means]

*EQ5D* EuroQoL-5D, *VAS* visual analog scale, *MLHFQ* Minnesota Living with Heart Failure Questionnaire

The MLHFQ scores (Table 5) revealed a significant improvement for both groups (− 9.65 points; 95% CI: 9.04–20.02), with significant differences compared to baseline scores (*p* <  0.001) and 2.02 points less than the obtained at month 1 after implant (reduction of 12.21%; *P* = 0.39). At the end of the study period, HM group experienced the better response with respect to month 0 (TM: − 4.4 vs. HM: − 15.75). At 6 months, there were no significant differences between the two groups (TM: 15.80 versus HM: 13.21; *p* = 0.97).

## Discussion

### Principal findings

Among the main results of the NORDLAND study, we found that, a) there was not significant differences in HRQoL assessed through EQ-5D utilities and VAS questionnaires administered at 1 and 6 months after implant between users included in the two groups of follow-up, b) in MLHF questionnaire were found significant differences in both groups between the enrolment and the end of follow-up period, c) safety was similar between both groups and no significant differences were found, d) that number of visits to hospital for both groups was similar and no significant differences were found.

### Health-related quality of life

Several studies [10, 16–18, 30] have assessed HRQoL in users with pacemakers. In these clinical trials, the SF-36 [16, 17, 30] and EQ5D [10, 18] questionnaires were used to assess HRQoL. Results were improved but no significant differences between both groups in either the effectiveness or the safety of TM and HM were found.

Coinciding with previous trials [16, 17, 30] there was not found significant improvement in health-related quality of life for both groups between the enrolment and the last month of the study. In other more recent studies [17, 18], users with pacemaker implants included in both groups reported significant improvements in HRQoL assessed through the EQ-5D [10] and SF-36 [18]. On the other hand, in NORDLAND study the MLHF questionnaire score was improved in the studied groups (21.78% vs. 54.42%) and at 6 months there were statistically significant differences between the groups. These results were similar to those obtained in other studies in which HRQoL questionnaires for specific heart-diseases such as MLHFQ [30], AQUAREL [18], Duke Activity Status Index (DASI) [10] were improved. In all the studies, similar results have been found regardless their research design, either randomized or non-randomized.

### Safety of tele-monitoring of pacemaker users

Previous studies [7, 10, 17, 30] showed a reduction in the percentage of in-office follow-up visits (31 to 58%). These results contrast with the obtained in the NORDLAND study where the visits to the hospital were 2% higher in TM group with respect to the other group (TM:1.24 vs. HM:1.17; *p* = 0.26). Additionally, in NORDLAND study, there were 98 transmissions scheduled for home patient (TM group) plus 10 extra ones (unscheduled transmissions).

The results on early detection of cardiovascular events and safety were similar to those obtained in previous studies [10, 16, 17, 31–35]. There were no found significant differences among the two groups with regards to cardiovascular events. The cumulative number of cardiovascular events was slightly higher in the TM (6%) than in the HM group (4.2%) although no statistically significant differences were found. This is relevant since it has been demonstrated that either a high burden of atrial fibrillation can be an important predictor of stroke and even mortality [5, 11, 13]. On the other hand, results found in previous studies showed that users included in TM group were more satisfied, the number of in-office visits to hospital was reduced, feeling safer, and being closely monitored by competent health care professionals with negligible impact of tasks to be fulfilled to comply with monitoring [5, 18, 36, 37] and although the number of studies on HRQoL and costs performed up to present on telemonitoring of pacemakers is quite reduced, preliminary studies suggest costs-saving [15]. A very reduced number of studies on effectiveness in pacemakers telemonitoring have used the HRQoL (EQ5D and SF-36) [10, 16, 17] questionnaires.

### The added value of the NORDLAND study

To our knowledge, the NORDLAND study is the first randomized clinical trial (RCT) evaluating HRQoL in the tele-monitoring of users with pacemakers. The RCT is the most scientifically rigorous method of hypothesis testing available [38], and is regarded as the gold standard trial for evaluating the effectiveness of interventions [39]. Therefore, the NORDLAND study provides an important input for future systematic reviews and clinical decision-making with regards to the use of tele-monitoring of users with pacemakers. In addition, to our knowledge, the NORDLAND study is first using the Minnesota Living with Heart Failure Questionnaire, which offers an innovative approach in the field of internet-based health technologies assessments.

### Strengths and limitations

Despite the relevant results obtained, the study has some limitations. First, it is a trial where patients and health staff knew the type of monitoring assigned, possibly this aspect may have influenced in their behavior. However, such influence has been decreased as much as possible since the NORDLAND study is a randomized trial. This ensures major evidence level, less chance of bias due to random selection of the groups and it might be repeatable and comparable with other studies. Second, the final size of patients included in this trial (with a limited number of pacemakers implants per year) made it difficult to detect differences between the two groups. The lack of a larger final sample size and the limited differences between groups may have reduced the real statistical power. However, the proximity between the results found in both groups in the analyses performed do not indicate significant differences. In addition, our study is a single centre study which may also be an advantage as different clinical practice among different hospitals sometimes makes it difficult to conduct multicenter studies.

Moreover, the comparison and generalization of results to other settings may be supported as participant characteristics were quite similar to those reported in previous studies [2–7, 10, 16, 17] in terms of age, gender, symptoms and indications.

Finally, it must be considered the phenomenon known as the Hawthorne effect [40, 41]. The fact that people tend to change their behavior when they are the targets of interest and attention, regardless of the specific nature of an intervention could be a limitation of our results. In such situations, patients become eager to please their physicians and make them feel successful. Additionally, patients wish to participate so that “good” results can be achieved in the study [40, 41].

## Conclusions

To conclude, data obtained in the NORDLAND study show that health-related quality of life in patients with pacemakers is improved 6 months after implant in both groups. No significant differences between tele-monitoring and in-office follow-up groups were found with regards to effectiveness in terms of health-related quality of life, feasibility, reliability, safety and number of visits to hospital. Finally, this randomized clinical trial provides a scientifically rigorous method to the field of health-related quality of life evaluations in users with pacemakers.

Future research in this area should focus on interventions based on quality of life perceived by the patients and costs of multi-center studies involving a long-term follow-up of users with the telemonitoring of pacemakers and other types of cardiovascular implantable electronic devices.

## Abbreviations

AE: Adverse events
AF: Atrial fibrillation
CRT: Cardiac resynchronization therapy
DDDR: Dual chamber pacemaker
EQ-5D: EuroQol-5D
HM: Hospital monitoring
HRQoL: Health-Related Quality of Life
ICD: Implantable cardioverter-defibrillator
*MLHFQ*: Minnesota Living with Heart Failure Questionnaire
PM: Pacemakers
RCT: Randomized clinical trial
TM: Tele-monitoring
VAS: Visual analogue scale
VVIR: Single chamber pacemaker

## References

[1] Epstein AE, JP DM, Ellenbogen KA (2008). ACC/AHA/HRS 2008 Guidelines for Device-Based Therapy of Cardiac Rhythm Abnormalities: a report of the American College of Cardiology/American Heart Association Task Force on Practice Guidelines (Writing Committee to Revise the ACC/AHA/NASPE 2002 Guideline Update for Implantation of Cardiac Pacemakers and Antiarrhythmia Devices) developed in collaboration with the American Association for Thoracic Surgery and Society of Thoracic Surgeons. J Am Coll Cardiol.

[2] Wilkoff BL, Auricchio A, Brugada J (2008). HRS/EHRA expert consensus on the monitoring of cardiovascular implantable electronic devices (CIEDs): description of techniques, indications, personnel, frequency and ethical considerations. Heart Rhythm.

[3] Cronin E, Varma N (2012). Remote monitoring of cardiovascular implanted electronic devices: a paradigm shift for the 21st century. Expert Rev Med Devices.

[4] Folino AF, Breda R, Calzavara P, Migliore F, Iliceto S, Buja G (2012). In-home controls of pacemakers in debilitated elderly patients. Geriatr Gerontol Int.

[5] Folino A, Breda R, Calzavara P (2013). Remote follow-up of pacemakers in a selected population of debilitated elderly patients. Europace.

[6] López-Villegas A, Catalán-Matamoros D, Robles-Musso E, Peiró S (2016). Workload, time and costs of the informal cares in patients with tele-monitoring of pacemakers. The PONIENTE study. Clin Res Cardiol.

[7] Perl S, Stiegler P, Rotman B (2013). Socio-economic effects and cost saving potential of remote patient monitoring (SAVE-HM trial). Int J Cardiol.

[8] Varma N, Ricci RP (2013). Telemedicine and cardiac implants: what is the benefit?. Eur Heart J.

[9] Osca J, Sanchotello MJ, Navarro J (2009). Technical reliability and clinical safety of a remote monitoring system for antiarrhythmic cardiac devices. Rev Esp Cardiol.

[10] López-Villegas A, Catalán-Matamoros D, Robles-Musso E, Peiró S (2015). Comparative effectiveness of remote monitoring of people with cardiac pacemaker versus conventional: quality of life at the 6 months. Rev Esp Salud Pública.

[11] Lopez-Villegas A, Catalan-Matamoros D, Robles-Musso E, Peiro S (2016). Effectiveness of pacemaker tele-monitoring on quality of life, functional capacity, event detection and workload: the PONIENTE trial. Geriatr Gerontol Int.

[12] Guédon-Moreau L, Lacroix D, Sadoul N, Clémenty J, Kouakam C, Hermida JS (2013). A randomized study of remote follow-up of implantable cardioverter defibrillators: safety and efficacy report of the ECOST trial. Eur Heart J.

[13] Ricci RP, Morichelli L, Santini M (2008). Home monitoring remote control of pacemaker and implantable cardioverter defibrillator patients in clinical practice: impact on medical management and health-care resource utilization. Europace.

[14] Halimi F, Cantu F (2010). Remote monitoring for active cardiovascular implantable electronic devices; a European survey. Europace.

[15] López-Villegas A, Catalán-Matamoros D, Martín-Saborido C, Villegas-Tripiana I, Robles-Musso E (2016). A Systematic Review of Economic Evaluations of Pacemaker Telemonitoring Systems. Rev Esp Cardiol (Engl Ed).

[16] Halimi F, Clémenty J, Attuel P, Dessenne X, Amara W on behalf of the OEDIPE trial investigators (2008). Optimized post-operative surveillance of permanent pacemakers by home monitoring: the OEDIPE trial. Europace.

[17] Mabo P, Victor F, Bazin P (2012). A randomized trial of long-term remote monitoring of pacemaker recipients (the COMPAS trial). Eur Heart J.

[18] Comoretto RI, Facchin D, Ghidina M, Proclemer A, Gregori D (2017). Remote control improves quality of life in elderly pacemaker patients versus standard ambulatory-based follow-up. J Eval Clin Pract.

[19] Knox L, Rahman RJ, Beedie C (2017). Quality of life in patients receiving telemedicine enhanced chronic heart failure disease management: a meta-analysis. J Telemed Telecare.

[20] United Nations Department of Economic and Social Affairs. Demographic Yearbook - 2016. https://unstats.un.org/unsd/demographic-social/products/dyb/dybsets/2016.pdf.

[21] Pong RW, Pitbaldo RJ (2001). Don’t take “geography” for granted! Some methodological issues in measuring geographic distribution of physicians. Canadian J Rural Med.

[22] DesMeules M, Pong R, Lagacé C (2006). How healthy are rural Canadians? An assessment of their health status and health determinants.

[23] Ministry of Finance, Norway Government. Statistics Norway 2015. https://web.archive.org/web/20150924125714/http://www.ssb.no/en/befolkning/statistikker/folkemengde/aar/2015-02-19?fane=tabell&sort=nummer&tabell=218738

[24] Telecardiology system BIOTRONIK. BIOTRONIK Home Monitoring. https://www.biotronik.com/en-de/products/home-monitoring.

[25] EuroQol Group (1990). EuroQol a new facility for the measurement of health-related quality of life. Health Policy.

[26] Thomas S. FDA Medical Device Development Tool (MDDT) Qualification Package for the Minnesota Living with Heart Failure Questionnaire (MLHFQ). 2017. https://djhurij4nde4r.cloudfront.net/attachments/files/000/000/483/original/MLHFQ_FDA_Medical_Device_Development_Tool_(MDDT)_Qualification_Package.pdf?1516113948.

[27] Hole T, Grundtvig M, Gullestad L, Flønæs B, Westheim A. Improved quality of life in Norwegian heart failure patients after follow-up in outpatient heart failure clinics: results from the Norwegian heart failure registry. Eur J Heart Fail. 2010;(11):1247–52. 10.1093/eurjhf/hfq156. 10.1093/eurjhf/hfq156 20847014

[28] Kularatna S, Byrnes J, Chan YK, Carrington MJ, Stewart S, Scuffham PA (2017). Comparison of contemporaneous responses for EQ-5D-3L and Minnesota living with heart failure; a case for disease specific multiattribute utility instrument in cardiovascular conditions. Int J Cardiol.

[29] Austin J, Williams WR, Hutchison S (2012). Patterns of fatigue in elderly heart failure patients measured by a quality of life scale (Minnesota living with heart failure). Eur J Cardiovasc Nurs.

[30] Blum K, Gottlieb SS (2014). The effect of a randomized trial of home telemonitoring on medical costs, 30-day readmissions, mortality, and health-related quality of life in a cohort of community-dwelling heart failure patients. J Card Fail.

[31] Lazarus A (2007). Remote, wireless, ambulatory monitoring of implantable pacemakers, cardioverter defibrillators, and cardiac resynchronization therapy systems: analysis of a worldwide database. Pacing Clin Electrophysiol.

[32] Neuzil P, Taborsky M, Holy F, Wallbrueck K (2008). Early automatic remote detection of combined lead insulation defect and ICD damage. Europace.

[33] Ricci RP, Morichelli L, Santini M (2009). Remote control of implanted devices through home monitoring technology improves detection and clinical management of atrial fibrillation. Europace.

[34] Spencker S, Coban N, Koch L, Schirdewan A, Muller D (2009). Potential role of home monitoring to reduce inappropriate shocks in implantable cardioverter defibrillator patients due to lead failure. Europace.

[35] Ricci RP, Morichelli L, D'Onofrio A (2013). Effectiveness of remote monitoring of CIEDs in detection and treatment of clinical and device-related cardiovascular events in daily practice: the HomeGuide registry. Europace.

[36] Schoenfeld MH, Compton SJ, Mead RH (2004). Remote monitoring of implantable cardioverter defibrillators: a prospective analysis. Pacing Clin Electrophysiol.

[37] Joseph GK, Wilkoff BL, Dresing T, Burkhardt J, Khaykin Y (2004). Remote interrogation and monitoring of implantable cardioverter defibrillators. J Interv Card Electrophysiol.

[38] Last JM (2001). A dictionary of epidemiology.

[39] Akobeng AK (2005). Principles of evidence based medicine. Arch Dis Child.

[40] Meneguin S, Machado César LA (2012). Motivation and frustration in cardiology trial participation: the patient perspective. Clinics.

[41] Fletcher RH, Fletcher SW (2006). Epidemiologia clinica: elementos essenciais. Artmed.

